# An investigation of the molecular characterization of the tripartite motif (TRIM) family and primary validation of TRIM31 in gastric cancer

**DOI:** 10.1186/s40246-024-00631-7

**Published:** 2024-07-09

**Authors:** Yixin Ding, Yangyang Lu, Jing Guo, Shuming Chen, Xiaoxi Han, Shibo Wang, Mengqi Zhang, Rui Wang, Jialin Song, Kongjia Wang, Wensheng Qiu, Weiwei Qi

**Affiliations:** 1 Department of Oncology, The Affiliated Hospital of Qingdao University, Qingdao, China; 2 Department of Medical Oncology, Department of Cancer Center, Peking Union Medical College Hospital, Chinese Academy of Medical Sciences, Beijing, China; 3 Department of Urology, Qingdao Municipal Hospital, Qingdao University, Qingdao, China

**Keywords:** TRIM family, TRIM31, Gastric cancer, Risk score, Prognostic biomarkers

## Abstract

Most TRIM family members characterized by the E3-ubiquitin ligases, participate in ubiquitination and tumorigenesis. While there is a dearth of a comprehensive investigation for the entire family in gastric cancer (GC). By combining the TCGA and GEO databases, common TRIM family members (TRIMs) were obtained to investigate gene expression, gene mutations, and clinical prognosis. On the basis of TRIMs, a consensus clustering analysis was conducted, and a risk assessment system and prognostic model were developed. Particularly, TRIM31 with clinical prognostic and diagnostic value was chosen for single-gene bioinformatics analysis, in vitro experimental validation, and immunohistochemical analysis of clinical tissue microarrays. The combined dataset consisted of 66 TRIMs, of which 52 were differentially expressed and 43 were differentially prognostic. Significant survival differences existed between the gene clusters obtained by consensus clustering analysis. Using 4 differentially expressed genes identified by multivariate Cox regression and LASSO regression, a risk scoring system was developed. Higher risk scores were associated with a poorer prognosis, suppressive immune cell infiltration, and drug resistance. Transcriptomic data and clinical sample tissue microarrays confirmed that TRIM31 was highly expressed in GC and associated with a poor prognosis. Pathway enrichment analysis, cell migration and colony formation assay, EdU assay, reactive oxygen species (ROS) assay, and mitochondrial membrane potential assay revealed that TRIM31 may be implicated in cell cycle regulation and oxidative stress-related pathways, contribute to gastric carcinogenesis. This study investigated the whole functional and expression profile and a risk score system based on the TRIM family in GC. Further investigation centered around TRIM31 offers insight into the underlying mechanisms of action exhibited by other members of its family in the context of GC.

**Supplementary Information:**

The online version contains supplementary material available at 10.1186/s40246-024-00631-7.

## Introduction

The prevalence and fatality rates of gastric cancer (GC) have become significant global concern in the realm of human health, demanding attention and action. With the increasing depth of exploration into the molecular process of GC, a growing number of molecular targets and biomarkers have been identified. Nevertheless, there remains a scarcity of targets possessing substantial pharmaceutical value, and the issue of drug resistance persists. Hence, further investigation into chemicals that hold prognostic and carcinogenic relevance can contribute to a more comprehensive comprehension of GC.

Gene families are established through a shared ancestral origin and demonstrate significant structural and functional resemblances, leading to the generation of related protein products [1]. Numerous gene families have been confirmed to be associated with the process of carcinogenesis [2, 3]. The tripartite motif (TRIM) family has more than 80 members and is primarily distinguished by the interesting new gene (RING) domain, which serves as an E3-ubiquitin ligase [4]. The TRIM family is known for its active involvement in various biological processes, including ubiquitination, regulation of immunological response, and cancer, owing to its distinctive domain [5]. Multiple studies have provided evidence indicating that the expression levels of TRIM family genes are associated with negative clinical outcomes in various types of cancer, including acute myeloid leukemia (AML), hepatocellular carcinoma (HCC), colorectal cancer (CRC), pancreatic cancer, breast cancer, lung cancer, and GC [6–12]. Nevertheless, there is a notable scarcity of extensive research investigating the combined manifestation of the TRIM family and integrating risk evaluation within the framework of GC.

The primary aim of our study was to examine the expression patterns and prognostic characteristics of TRIM family members in GC. Furthermore, gene clusters were obtained through the utilization of consensus clustering analysis, with particular attention given to the expression patterns exhibited by members of the same family. In addition, a risk score pertaining to TRIM was created in order to assess discrepancies in characteristics such as immune infiltration, medication sensitivity, and prognosis between groups classified as high-risk and low-risk. Moreover, the selection of TRIM31, a member of this gene family that demonstrates prognostic significance, was made to undertake comprehensive investigation using bioinformatic analysis and in vitro experiments.

## Results

### Expression and survival analyses of TRIMs

**Fig. 1:**
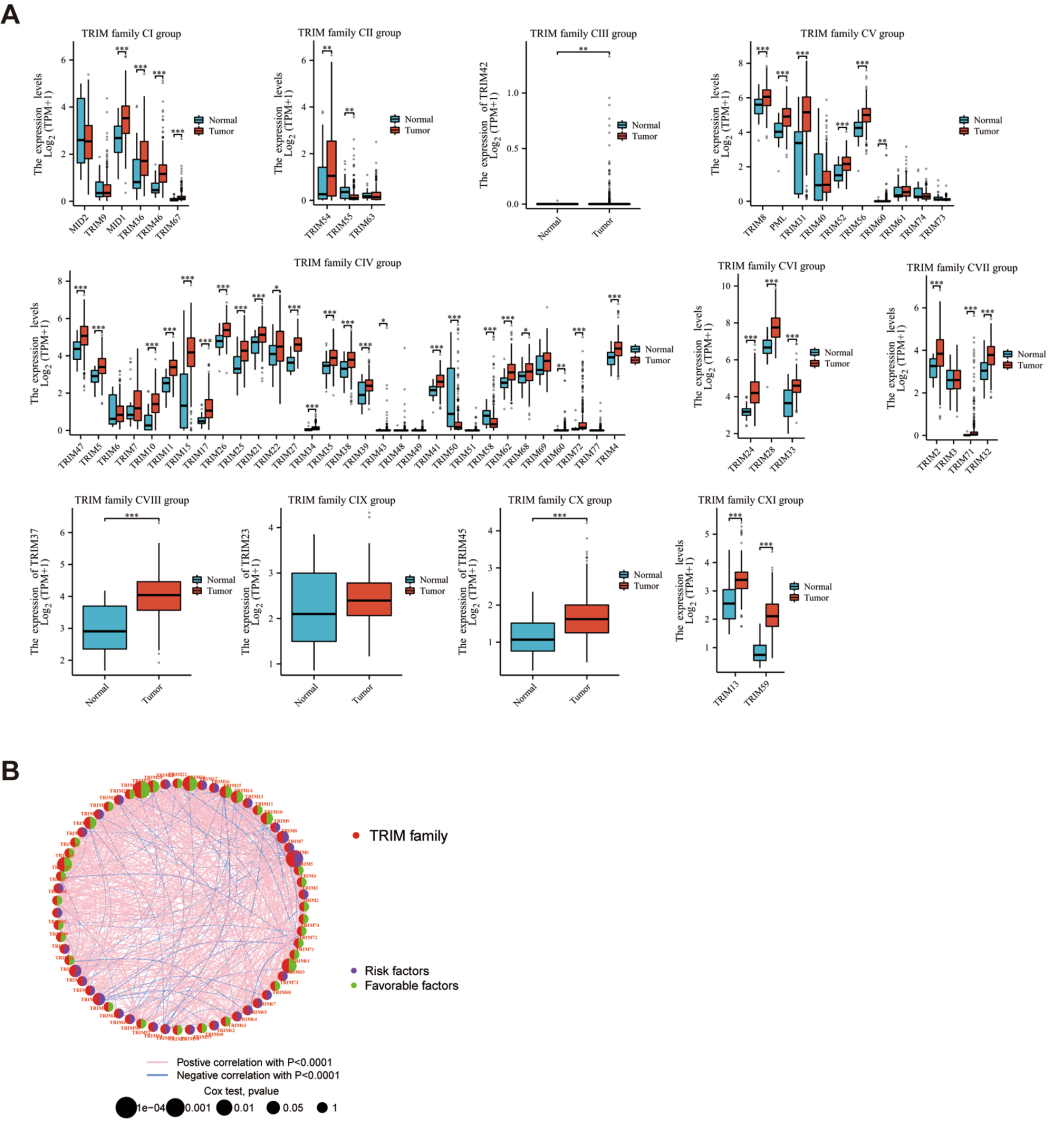
mRNA expression profile and prognostic network of TRIMs. (**A**) The expression level of TRIM family CI-CXI groups between gastric STAD and normal tissues. MID1: TRIM18. MID2: TIRM1. PML: TRIM19. (**B**) The prognostic network diagram was drawn according to the results of Univariate Cox regression analysis between TRIM family members and OS of STAD. HR > 1 was defined as risk factors, and HR < 1 was favorable factors. ns, *p* ≥ 0.05; *p < 0.05; **p < 0.01; ***p < 0.001

The merged RNA-Seq transcriptome data was defined as merged-matrix, which concluded 63 TRIMs. The expression level of TRIMs were displayed between STAD and normal samples from TCGA (Fig. 1A). The prognostic network diagram of the univariate Cox regression analysis of TRIMs was showed in Fig. 1B. There were 52 DEMs expressed differentially in STAD merged-matrix. The expression levels of 44 DEMs were significantly correlated with the OS according to the K-M analysis (Supplementary Figs. 1–2).

### Gene alteration profile, TF-gene-miRNA and PPI network of TRIMs

**Fig. 2:**
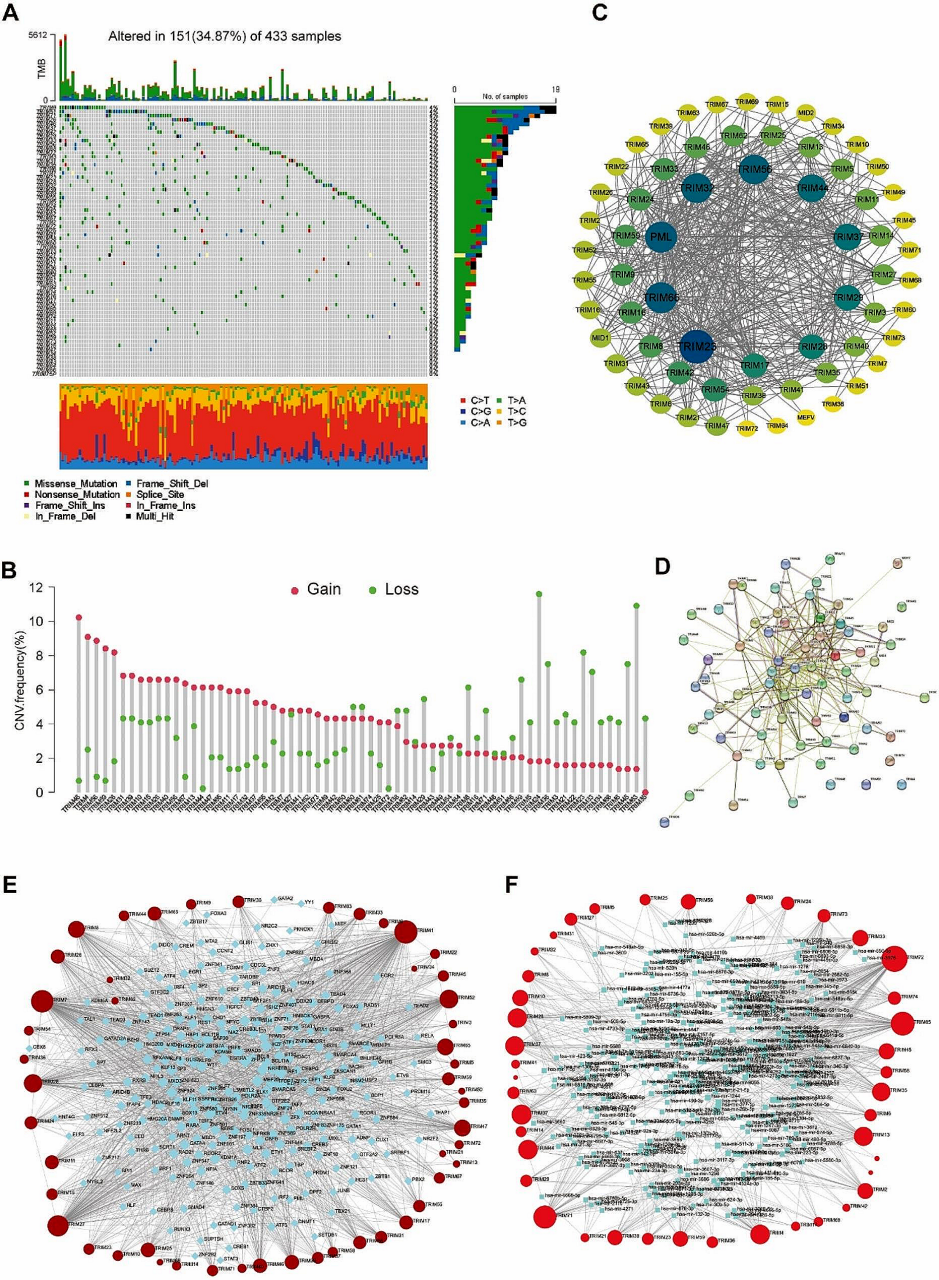
Gene mutation profile and interaction networks of TRIMs. (**A**) Visualization of the somatic mutation profile of TRIMs from 433 samples of TCGA-STAD. Genes are ordered by their mutation frequencies. The mutation frequency and number of samples for each gene are shown on the right. The bottom side shows information about the nucleic acid mutation sites and the type of mutation. (**B**) The CNV frequency of TRIMs. The values represented by the red circles indicated the gain frequency of the gene, while the green circles indicated the loss frequency. (**C**) The PPI network of TRIMs used Cytoscape software. The ordering of “Degree” is quantized using the size and color of the circle. Larger circles and darker colours represent stronger interactions. (**D**) The network of functional and physical protein associations of TRIMs by the STRING database with 0.400 interaction score. (**E**) The TF-gene interaction network constructed by the ENCODE database with the peak intensity signal < 500 and the predicted regulatory potential score < 1. Red circles represent TRIMs, blue squares represent transcription factors. The size of the red circle was positively related to the number of interacted TFs. (**F**) The gene-miRNA interaction network by the miRTarBase database. Red circles represent TRIMs, blue squares represent miRNAs. The size of the red circle was positively related to the number of interacted miRNAs.

The alteration frequency of 7 TRIMs were above 2%, including TRIM9, TRIM51, TRIM71, TRIM26, TRIM46, TRIM37, and TRIM42. Missense mutation accounted for the majority of gene alteration (Fig. 2A). The increased copy number was discovered in 37 TRIMs, while the decreased copy number was discovered in 28 TRIMs (Fig. 2B). The PPI network of proteins coded by TRIMs was showed in Fig. 2C-D. Figure 2E and F displayed the TF-gene and gene-miRNA network of TRIMs, respectively.

### Consensus clustering analysis of TRIMs

**Fig. 3:**
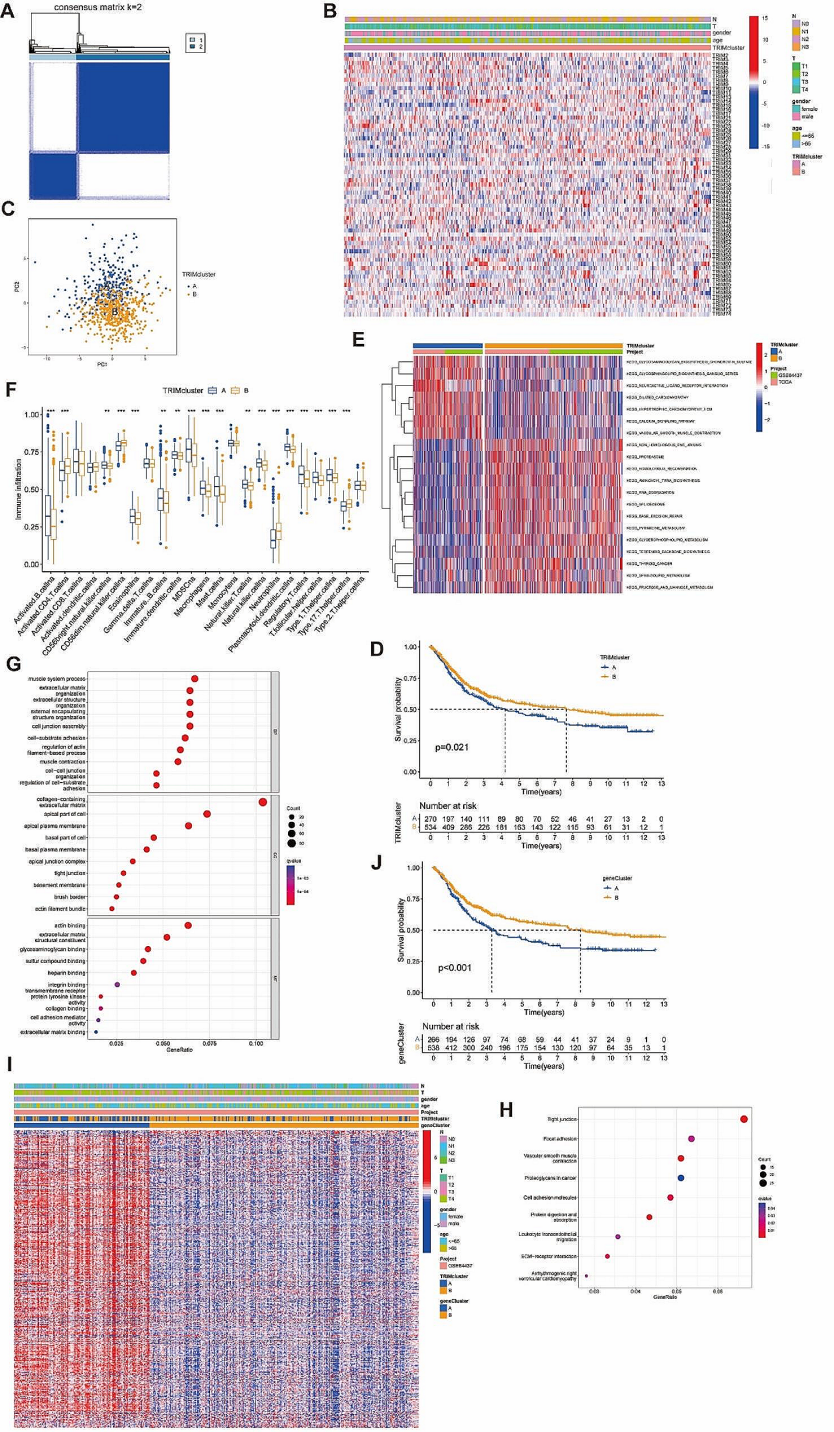
The consensus clustering analysis and functional analysis. (**A**) When the value of k (representing the number of clusters) is set to 2, the consensus matrix is generated to describe the outcomes of the clustering. (**B**) Heatmap of the expression of TRIMs in TRIM cluster A and B with clinical characteristics (N stage, T stage, gender and age). (**C**) PCA of TRIM cluster A and B of GC patients. Visualization by the PCA analysis, mapping from high dimensions to two dimensions. (**D**) K-M analysis between TRIM cluster A and B. (**E**) The different enriched pathways between TRIM cluster A and B by the GSVA analysis. Enriched pathways were shown on the right. (F) Immune infiltration score of immune cells in TRIM cluster A and B by the ssGSEA analysis. The horizontal coordinates represented the different types of immune cells and the vertical coordinates represented the corresponding immune infiltration scores. GO (**G**) and KEGG (**H**) enrichment analyses conducted in the DEGs. The size of the circle represented the degree of enrichment. BP: Biological Process. MF: Molecular Function. CC: Cellular Component. (**I**) Heatmap of the expression of TRIMs in gene cluster A and B after the second consensus clustering analysis with clinical characteristics (N stage, T stage, gender and age). (**J**) K-M analysis between gene cluster A and B. ns, *p* ≥ 0.05; *p < 0.05; **p < 0.01; ***p < 0.001

The optimal K value was determined to be K = 2 according to the consensus clustering analysis (Fig. 3A, Supplementary Fig. 3A-G, Supplementary Fig. 5A). STAD samples were divided into TRIM cluster A and TRIM cluster B (Fig. 3B). PCA analysis showed favorable discrimination between the two clusters (Fig. 3C). K-M analysis indicated better survival benefit of TRIM cluster B than cluster A (*p* = 0.021) (Fig. 3D). GSVA analyses showed glycosaminoglycan biosynthesis chondroitin sulfate, neuroactive ligand receptor interaction pathways were more enriched in cluster A. While proteasome, aminoacyl tRNA biosynthesis, RNA degradation, spliceosome, base excision repair were more enriched in cluster B (Fig. 3E). ssGSEA analysis showed that a variety of immune cells were more enriched in TRIM cluster A, including activated B cells, mature B cells, CD56 bright natural killer (NK) cells, MDSC, macrophage, mast cell, natural killer T cells, NK cells, plasmacytoid dendritic cells (DCs), T follicular helper cells (TFH), and Th1 cells. While, activated CD4 T cells, CD56 dim NK cells, neutrophil, and Th17 cells were more enriched in TRIM cluster B (Fig. 3F).

818 DEGs were obtained from the differential expression analysis between TRIM cluster A and B. GO and KEGG enrichment analysis showed DEGs were most enriched in components and structure of extracellular matrix (Fig. 3G-H). Gene cluster A and B were obtained by consensus clustering analysis based on DEGs (Fig. 3I, Supplementary Fig. 3A-G, Supplementary Fig. 5B). Gene cluster A and B exhibited more pronounced prognostic disparities in comparison to TRIM cluster A and B (*p* < 0.001) (Fig. 3J). 38 TRIMs were differentially expressed between the two gene clusters (Supplementary Fig. 5C).

### Prognostic model construction and scRNA-seq analysis based on hub DEGs

**Fig. 4:**
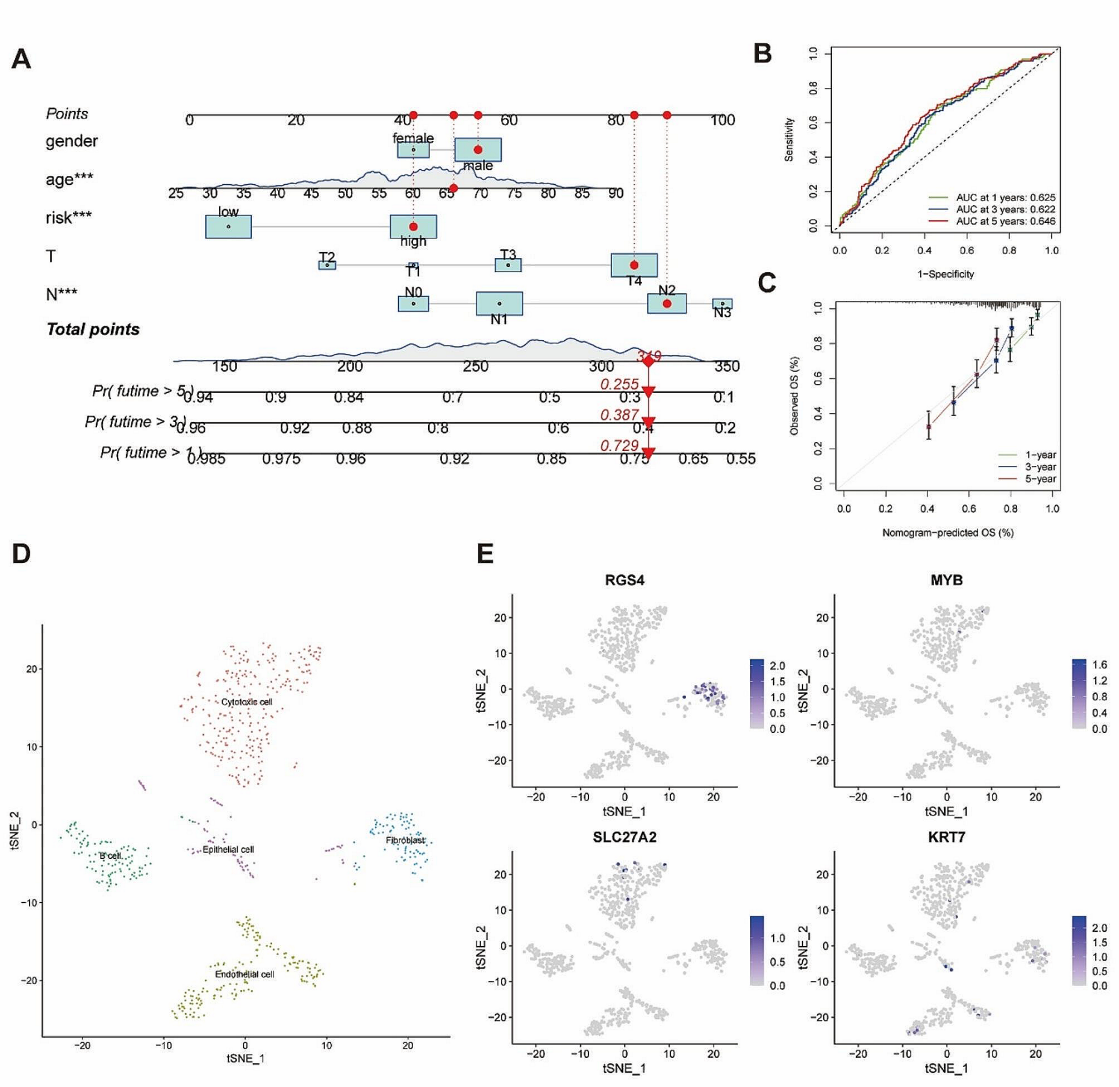
The nomogram based on the risk score and scRNA analysis of hub genes. (**A**) The survival rate at 1, 3, and 5-year of a sample from TCGA-STAD were 0.729, 0.387 and 0.255, respectively, according to the nomogram based on gender, age, risk score, T stage and N stage. (**B**) The ROC of the model and AUC at 1, 3, and 5-year. (**C**) Calibration curve of the model at 1, 3, and 5-year. (**D**) The t-SNE algorithm was used for dimensionality reduction and 5 cell subgroups were identified. (**E**) The expression of hub genes in 5 cell subgroups. ns, *p* ≥ 0.05; *p < 0.05; **p < 0.01; ***p < 0.001

7 DEGs were screened out by the LASSO regression, including RGS4, MYB, EDNRA, SLC27A2, TAGLN, HTRA1, and KRT7 (Supplementary Fig. 5D). 4 hub DEGs including RGS4, MYB, SLC27A2, and KRT7 satisfied the criteria of the multivariate Cox regression with p value < 0.05 and were selected as the model constituent genes. STAD samples from the merged-matrix were divided into a train set and a test set at a 1:1 ratio, and the train set was used to constructed the Cox regression model and nomogram (Fig. 4A). The calibration curve was close to the diagonal and the AUC of 1-, 3-, and 5-years ROC curve were 0.625, 0622, and 0.646 in all samples (Fig. 4B-C). The ROC curve with AUC of the predicted model was also obtained in train set and test set at 1, 3, and 5-year (Supplementary Fig. 5E-F).

The scRNA-seq data of our study was obtained from the GSM5101015 diffuse-type GC sample with 2234 cell samples. After unsupervised clustering, cell annotation and visualization of clusters, we identified 7 clusters as 5 kinds of cell subgroups: Cytotoxic cell, Endothelial cell, B cell, Fibroblast and Epithelial cell (Fig. 4D). RGS was verified expressed mainly in fibroblast cell subgroup (Fig. 4E).

### High risk group displayed unfavorable survival outcome and impressive immune cell infiltration

**Fig. 5:**
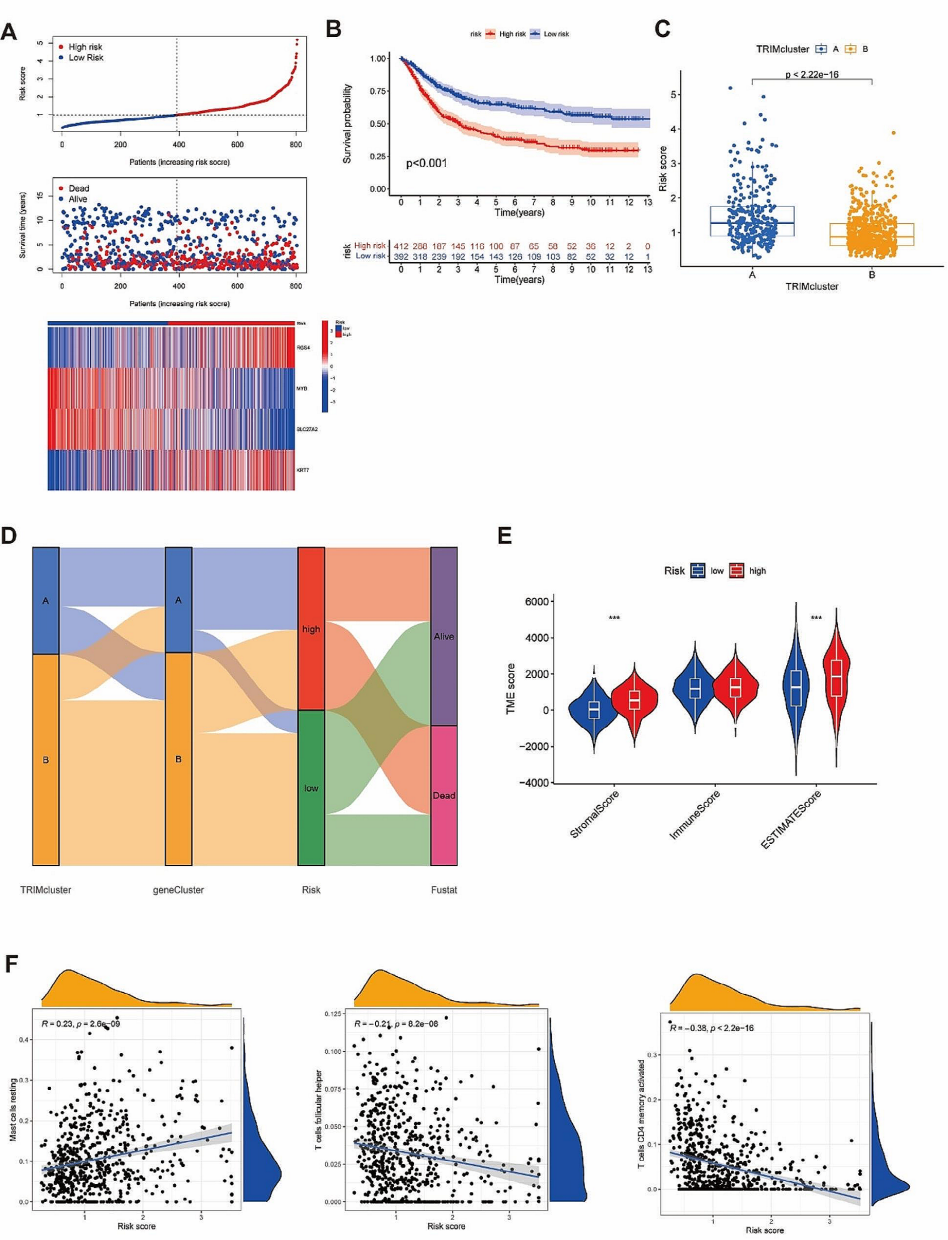
Survival analysis and immune infiltration analysis between high-risk and low-risk groups. (**A**) The risk score, survival time and expression level of hub genes in high-risk and low-risk groups in all samples. (**B**) K-M analysis between high-risk and low-risk groups. (**C**) The risk score of TRIM cluster A was higher than TRIM cluster B. (**D**) The Sankey diagram displayed the distribution of the samples after twice consensus clustering analysis and risk score construction, as well as survival outcomes. (**E**) Stromal score, immune score and ESTIMATE score in high-risk and low-risk groups. (**F**) The correlation analysis in CD4 + memory T cells, mast cells and Tfh with risk score. R referred as the correlation coefficient. ns, *p* ≥ 0.05; *p < 0.05; **p < 0.01; ***p < 0.001

The formula of risk score was displayed as: risk score =(0.206021*RGS4) + (-0.09742*MYB) + (-0.24672*SLC27A2) + (0.107758*KRT7). ALL STAD samples were divided in to low- or high-risk group based on the median of risk score (Fig. 5A, Supplementary Fig. 6A-B). K-M analysis showed better survival in low-risk group (*p* < 0.001) (Fig. 5B, Supplementary Fig. 6C-D). 36 genes in TRIMs were differentially expressed between the two groups, and TRIM cluster A and gene cluster A had higher risk scores (*p* < 0.001) (Fig. 5C, Supplementary Fig. 6E). Sankey diagram showed the distribution of STAD samples between TRIM clusters, gene clusters, risk groups and clinical outcomes (Fig. 5D).

The stromal score and ESTIMATE score were higher in the high-risk group (Fig. 5E). Immune cell infiltration analysis showed the infiltration of resting mast cells, memory B cells, M2, monocytes, resting memory CD4 + T cells and Tregs were positively related to risk scores, while activated memory CD4 + T cells, TFHs, M0, M1, resting NK cells, and plasma cells were negatively related to risk scores. In particular, resting mast cells, activated memory CD4 + T cells, and TFHs were closely related to risk scores (|Spearman correlation coefficient| >0.2) (Fig. 5F, Supplementary Fig. 5F).

### High risk group was connected with MSS/MSI-L status and higher IC50 of drugs

**Fig. 6:**
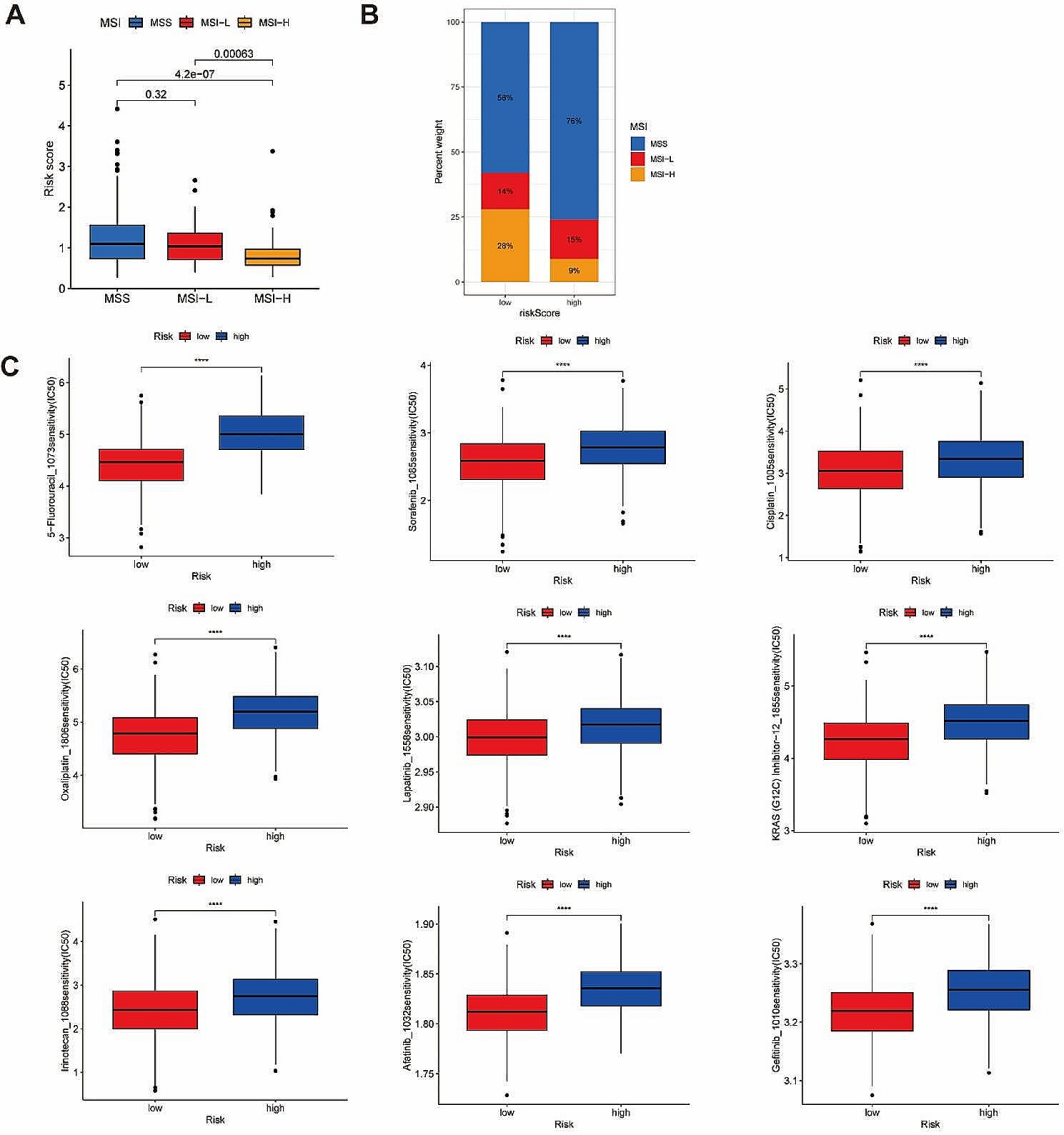
Exploration of MSI status and drug sensitivity between high-risk and low-risk groups. (**A**) The relationship between MSI status and risk score. The risk score was with significant statistical difference between MSS and MSI-H, MSI-L and MSI-H. (**B**) The percent weight of different MSI status in high-risk and low-risk group. (**C**) The IC50 of 9 kinds of drugs in high-risk and low-risk group. ns, *p* ≥ 0.05; *p < 0.05; **p < 0.01; ***p < 0.001

Patients belong to the low-risk group had the tendency to present MSI-H status (Fig. 6A-B). Significantly statistical difference of IC50 of multiple drugs were displayed between high and low risk group. Higher IC50 of drugs (chemotherapy drugs: 5-Fluorouracil, cisplatin, cyclophosphamide, docetaxel, oxaliplatin, gemcitabine, irinotecan, paclitaxel, and so on; targeted drugs: afatinib, dabrafenib, erlotinib, gefitinib, lapatinib, sorafenib, and so on) was found in high group (Fig. 6C).

TRIM31 was up-expressed on gastric tissues and indicated unfavorable clinical prognosis.

**Fig. 7:**
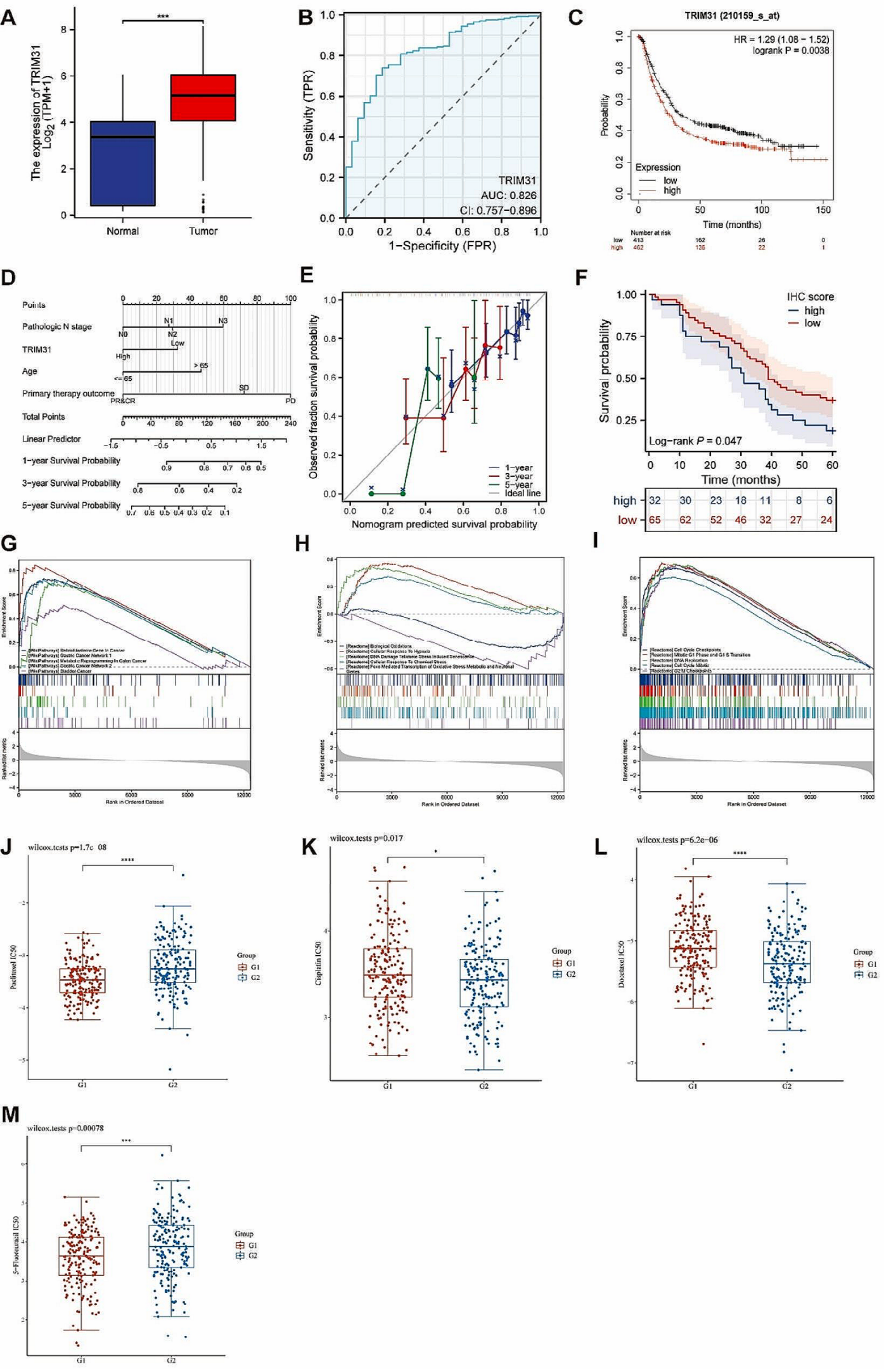
Single-gene bioinformatics analyses of TRIM31. (**A**) The expression level of TRIM31 between STAD and normal tissue. (**B**) The ROC curve with AUC of the accuracy of TRIM31 for predicting outcomes in STAD. (**C**) K-M analysis between TRIM31-high and TRIM31-low groups. (**D**) The 1-, 3-, and 5-year nomogram was constructed based on independent risk factors of OS after Cox regression. (**E**) Calibration curve of the model at 1, 3, and 5-year. (**F**) K-M analysis between high TRIM31 IHC score and low score groups. (**G**-**I**) GSEA analysis on DEGs between TRIM31-high and TRIM31-low groups. (**J**-**M**) The IC50 of paclitaxel, cisplatin, docetaxel and 5-fluorouracil in TRIM31-high (G1) and TRIM31-low (G2) groups. ns, *p* ≥ 0.05; *p < 0.05; **p < 0.01; ***p < 0.001

We found that the TRIM31 was overexpressed in STAD compared with the normal and paracancerous tissues (Fig. 7A). Patients with distance metastasis displayed higher TRIM31 expression (Supplementary Fig. 7A). The ROC curve indicated the favorable identification ability in GC of TRIM31 (Fig. 7B). In STAD samples, the OS of TRIM31 high expression group was better than that of TRIM31 low expression group (Fig. 7C). The N stage, age, primary therapy outcome and the expression level of TRIM31 were proved to be the independent prognostic risk factor of the multivariate Cox regression analysis, and were included to construct the prognostic model and the nomogram (Fig. 7D-E).

The IHC score of STAD samples were significantly higher than paracancerous samples in tissue microarray chips (Table 1). Clinical characteristics of STAD patients in tissue microarray chips was shown in (Supplementary Tabel 1). TRIM31-low group was verified with longer survival time than TRIM31-high group based on the ICH score in STAD samples, the mOS was 39 m (95%CI 35-NA) and 31 m (95%CI 26–42) (*P* = 0.0471), respectively (Fig. 7F). IHC score of TRIM31, age, T stage and N stage were verified to be correlated with the overall survival of STAD patients in univariate Cox regression analysis and were submitted to multivariate analysis (Table 2).

**Table 1:** Differential expression of TRIM31 in cancer and paracancerous tissues

Group	n	TRIM31 expression		χ2	p value
		High	Low		
Cancer tissues	97	32	65	12.47	0.000
Paracancerous tissues	83	9	74		

**Table 2:** Univariate and multivariate analyses of the factors correlated with Overall survival of STAD patients

Characteristics	Univariate analysis			Multivariate analysis	
	Hazard ratio (95% CI)	*P* value		Hazard ratio (95% CI)	*P* value
IHC score of TRIM31					
low	Reference			Reference	
high	1.642 (1.003–2.689)	0.049		0.947 (0.555–1.614)	0.840
Sex					
Male	Reference				
Female	1.070 (0.617–1.857)	0.809			
Age	1.030 (1.004–1.057)	0.022		1.023 (0.996–1.051)	0.102
T stage					
T1	Reference			Reference	
T2	1.441 (0.373–5.574)	0.596		1.242 (0.318–4.843)	0.755
T3	3.515 (1.076–11.486)	0.037		2.371 (0.702–8.003)	0.164
T4	9.291 (2.754–31.351)	< 0.001		5.546 (1.537–20.009)	0.009
N stage					
N0	Reference			Reference	
N1	0.680 (0.266–1.737)	0.420		0.834 (0.323–2.152)	0.708
N2	1.547 (0.788–3.040)	0.205		1.361 (0.674–2.749)	0.390
N3	2.793 (1.492–5.228)	0.001		2.097 (1.061–4.145)	0.033
Pathological grade					
Well differentiated	Reference				
Moderately differentiated	1.468 (0.440–4.894)	0.532			
Poorly differentiated	2.237 (0.691–7.247)	0.179			
Hp					
positive	Reference				
negative	0.989 (0.571–1.714)	0.970			
unknown	1.088 (0.583–2.028)	0.791			

### Deep research on TRIM31 based on bioinformatics analysis

7 mi RNAs and 25 TFs were verified to have interaction with TRIM31 (Supplementary Fig. 7B). DEGs between the TRIM31-high and TRIM31-low group were selected with the threshold mentioned before. 4424 positively and 1478 negatively co-expressed genes of TRIM31 were selected and the top 300 co-expressed genes were used to construct the PPI network (Supplementary Fig. 7C). We identified the top 9 enrichment items of BP, CC, MF, and KEGG pathways (Supplementary Fig. 7D). Cell cycle, oxidative stress and cell stress, and tumorigenesis related pathways were verified to be strongly connected with TRIM31 by GSEA analysis (Fig. 7G-I, Supplementary Fig. 7E). Especially, lower expression of TRIM31 was related with the activation of FOXO mediated transcription of oxidative stress. Moreover, immune cells including aDC, mast cells, NK cells, pDC, T helper, and Treg were positively related to TRIM31, while the stomal score was negatively related to TRIM31 (Supplementary Fig. 7F-G). Finally, we evaluated the relationship of the sensibility of GC common chemotherapy drugs and the expression of TRIM31. IC50 of 5-Fluorouracil and paclitaxel were negatively related to TRIM31, while cisplatin and docetaxel were positively related to TRIM31 which indicated that the expression of TRIM31 may serve as a biomarker of sensibility of chemotherapy in GC patients (Fig. 7J-M).

### Knockdown of TRIM31 inhibited the growth of GC cells

**Fig. 8:**
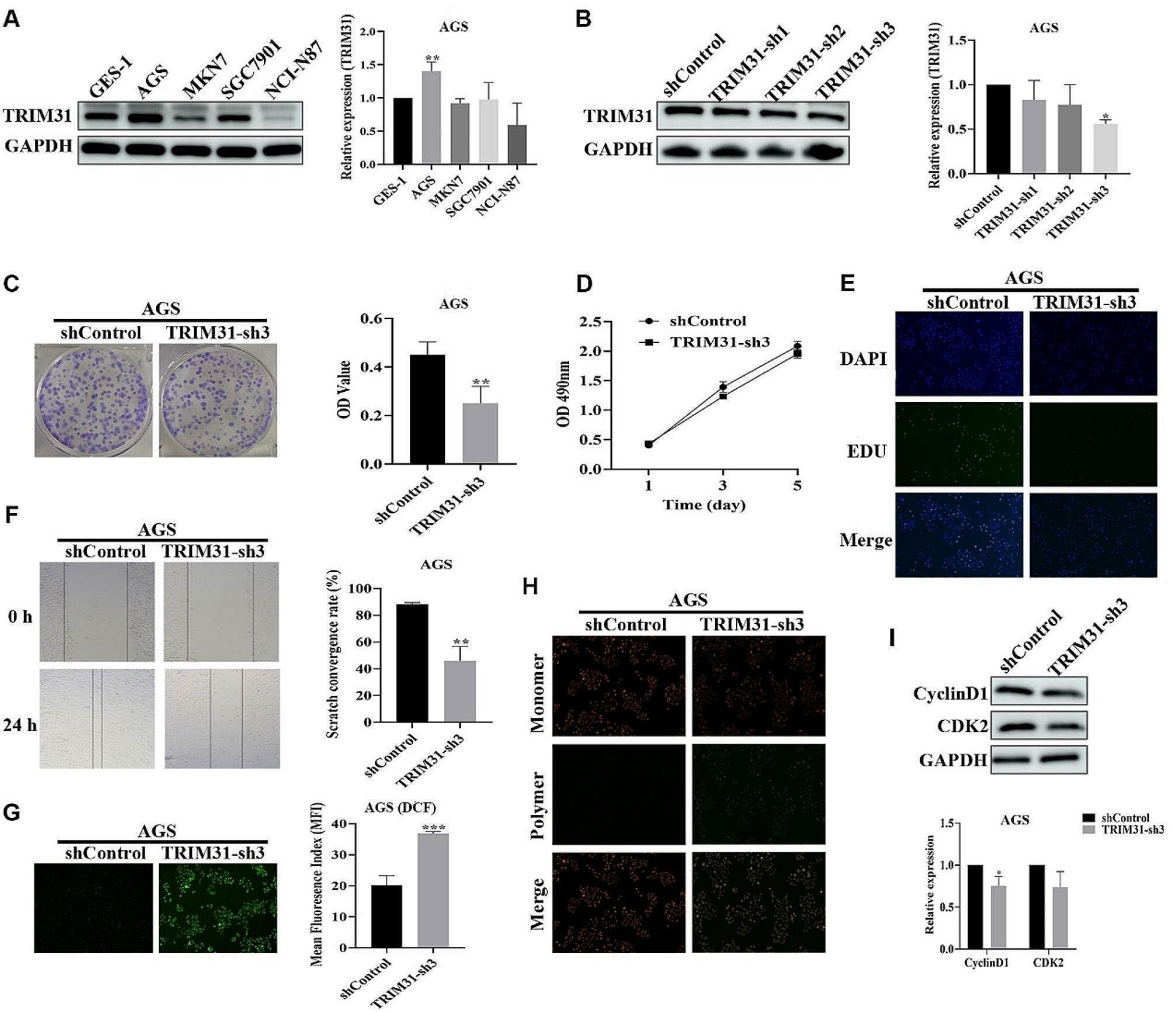
Functional validation of TRIM31 in vitro experiments. (**A**) Western blot revealed TRIM31 expression levels in GES-1, AGS, MKN7, SGC7901, NCI-N87 cell lines (*n* = 3). (**B**) TRIM31 protein expression was evaluated by western blot in AGS cells silenced by TRIM31-sh1, TRIM31-sh2, and TRIM31-sh3 (*n* = 3). (**C**) AGS cell lines cloning after lentivirus treatment (shControl, TRIM31-sh3). The absorbance of the solution generated by dissolving the cell population in glacial acetic acid represents the number of cell clones (*n* = 4). (**D**) Cell viability of AGS cell lines treated with lentivirus (shControl, TRIM31-sh3) was determined (*n* = 3). (**E**) EDU staining was used to detect the potential of AGS cells to proliferate. Scale bar: 100 μm. (**F**) Determination of migration ability of AGS cell lines treated with lentivirus (shControl, TRIM31-sh3) (*n* = 3). Scale bar: 100 μm. (**G**) Evaluate the oxidative stress level of AGS cell lines treated with lentivirus (shControl, TRIM31-sh3) by DCFH-DA staining (*n* = 3). Scale bar: 100 μm. (H) Evaluate the mitochondrial function of AGS cell lines treated with lentivirus (shControl, TRIM31-sh3) by JC-1 staining. Scale bar: 100 μm. (**I**) Western blotting was used to assess CyclinD1 and CDK2 levels in AGS cell lines treated with lentivirus (shControl, TRIM31-sh3) (*n* = 3). ns, *p* ≥ 0.05; *p < 0.05; **p < 0.01; ***p < 0.001

We know that TRIM31 is highly expressed in GC tissue based on prior immunohistochemistry data. We further look into the biological function of TRIM31 in GC via in vitro research. First, we compared the levels of TRIM31 protein expression in GES-1, AGS, MKN7, SGC7901, and NCI-N87 cell lines. The findings revealed that the protein expression level of TRIM31 in AGS cells was significantly higher than in the GES-1 cells (Fig. 8A). As a result, we chose AGS cells as the experimental subjects in the following investigations.

We are going to inject the lentivirus into AGS cells in order to create a stably transfected cell line with decreased TRIM31 protein. Western blot was utilized to test the efficacy of lentivirus infection, and it was discovered that the decrease in TRIM31 expression in TRIM31-sh3 AGS cells was the most significant (Fig. 8B). Following that, we assessed the proliferative potential of TRIM31-sh3 AGS cells and discovered that TRIM31 knockdown resulted in a decrease in cell proliferative ability (Fig. 8C). The similar result was observed when we utilized EDU staining to measure cell proliferation ability (Fig. 8E). It is worth mentioning that the reduction in cell viability caused by TRIM31 knockdown is not considerable (Fig. 8D). Additionally, we assessed the TRIM31-sh3 AGS cells’ capacity for migration and discovered that this capacity declined after TRIM31 knockdown (Fig. 8F). The rise in ROS levels in cells following TRIM31 knockdown, a sign of increased oxidative stress, is exciting (Fig. 8G). The data from JC-1 staining further revealed that TRIM31 knockdown impacted the mitochondrial activity of cells (Fig. 8H). Furthermore, western blot analysis revealed a decrease in CyclinD1 and CDK2 protein expression in TRIM31 knockdown cells (Fig. 8I).

## Discussion

The TRIM family proteins were characterized as the N-terminal RING domain, B-box domain, and a CC domain, of which the dimerization of RING domain was crucial to the activation of E3 ubiquitin ligases [13]. Comprising a large protein family, the members are divided in to C-I to C-XI based on the various C-terminus of TRIM protein [5, 14]. Thus lead to the diverse and even diametrically opposite cell function in cell proliferation and mitosis, immune response, tumorigenesis or harmonization of certain ubiquitination-related signal pathways [15–17]. Studies have verified that abnormal expression of TRIM family members were existed in multiple cancers [6, 17–19], and effected the tumor cells by promoting epithelial mesenchymal transition (EMT) and aerobic glycolysis [20]. The TRIM family functioned in tumorigenesis based on their other non-classical domains. TRIM14 and TRIM21 regulated the nuclear factor-κB (NF-κB) and phosphoinositide 3-kinase (PI3K)/protein kinase B (AKT) pathways by the PRYSPRY domain [21, 22]. TRIM44 was verified to be participated in the resistance of cisplatin in lung adenocarcinoma via deubiquitinating the K48-linked polyubiquitin chain with the ZnF UBP domain [23].

The unsupervised consensus clustering analysis provided with a more effective, accurate and visible method to classify disease phenotypes [24]. After doing the consensus clustering analysis, we identified differentially expressed genes (DEGs) from the two gene clusters, namely TRIM clusters A and B. The process involved the examination of all mRNA-encoding genes acquired from the TCGA and GEO gastric cancer databases. The purpose was to investigate the genes that are linked to different gene clusters. Additionally, we aimed to enhance the model using LASSO regression, specifically the risk score system, which was seen as an enhanced abstraction of the various expression profile of the entire TRIM family. The utilization of consensus clustering and LASSO analysis has successfully discovered hub genes implicated in carcinogenesis or cancer inhibition [25], which indirectly supported the significance of the analysis for the entire TRIM family. RGS proteins induced the regulation of vimentin and E-cadherin, and involved in the process of tumor metastasis [26, 27]. Especially, the scRNA-seq study revealed a considerable upregulation of RGS4 in the fibroblast population, hence playing a crucial role in tumour invasion and the modulation of the extra-tumoral stroma.

The remarkable function of TRIM family in tumor immune escape by multiple signaling pathways (including the PI3K/AKT signaling pathway, NF-κB signaling pathway and Wnt signaling pathway) was also be verified in various studies [12, 28–30]. Furthermore, the tolerance and suppression of anti-tumor immune response via abnormal expression of immune checkpoint such as PD-1/PD-L1 in tumor immune microenvironment (TME) largely contributes the tumor immune escape [31].TRIM16 influenced the expression of PD-L1 by regulating the JAK/STAT signaling pathway in non-small cell lung cancer, and TRIM28 was related to the resistance of cytotoxic T lymphocyte-associated antigen-4 (CTLA-4) blockade by the ubiquitination of AMP-activated protein kinase in melanoma [32, 33]. Our study found that the TRIM cluster A that with a worse survival outcome was related with higher infiltration of MDSCs and Tregs. Risk score was positive related with the infiltration of M2 and Tregs, while negatively related with the activated CD4 + memory cells. In TME, regulatory T cells (Tregs) induces non-antigen specific suppressive immune response and myeloid-derived suppressor cells (MDSCs) are impaired in maturation and negatively regulate the immune response [34].Furthermore, M2 macrophages have been found to be linked with the restriction of immunological response, the promotion of angiogenesis, and the facilitation of pro-tumorigenic activity [35]. The expression of TRIM family in macrophages was verified to influence the interaction between macrophages and tumor cells, thus decrease the monitory effect on cancer cells [36, 37].

In our study, TRIM31 exhibited significant upregulation in gastric cancer tissues, and this elevated expression was correlated with a poorer prognosis. Furthermore, TRIM31 exhibited differential expression in gene clusters A and B. As a member of E3 ubiquitin ligase family, TRIM31 was characterized with RING finger domain and was found to be engaged in inflammations, cell cycle and oncogenesis [38]. The suppression of miR-876-3p/TRIM31 axis was related to the impeded AML cell growth, indicated the role of TRIM31 in tumor cell cycle [39]. The role of TRIM31 in regulation of tumorigenesis was related to various cell signaling pathways in different cancers. High-expressed TRIM31 was found in gallbladder cancer, pancreatic cancer, and colorectal cancer [40, 41] while low-expressed in breast cancer [42]. The overexpression of TRIM31 was demonstrated as a tumor suppressor that inhibited the proliferation of lung cancer and ovarian cancer cells [43, 44]. However, TRIM31 exhibits contradictory functions in the progression of cancer. In gallbladder cancer and glioma, TRIM31 played a carcinogenic role by activating PI3K-Akt pathway and the resistance of chemical agents [45, 46]. In hepatocellular carcinoma, TRIM31 was related to the over activation of AMPK pathway by suppressing P53, which was the tumor suppressor protein [47].

Cell stress (including oxidative stress and inflammation responses) play a crucial role in oncogenesis by regulating Wnt, NF-κB and TGF-β related signaling pathways [48]. Multiple cytokines induced the activation of NF-κB pathway, thus contributed to the enhance of inflammation response, EMT and tumorigenesis. Ge et al. found that the function of Mulberrin against hepatic fibrosis and oxidative stress was depended on the expression of TRIM31 and the suppression of NF-κB pathway and NOD-like receptor protein 3 (NLRP3) inflammasome [49]. In pancreatic cancer and colorectal cancer, TRIM31 played a role in the activation of the NF-κB and the downstream genes [41, 50]. Moreover, downregulated TRIM31 elevated the level of reactive oxygen species (ROS) and strengthened the apoptosis in colorectal cancer cells [51]. Pathway enrichment analyses verified high expression of TRIM31 was involved in GC networks, related to cell cycle and DNA replication. The activation of cellular response to hypoxia and chemical stress may be reliant on high expression of TRIM31, therefore suggesting its indispensability. While, suppression of TRIM31 expression may trigger oxidative stress in tumor cells, leading to apoptosis. The results of out in vitro experiments have validated the significant conclusions derived from the pathway enrichment analysis. The knockdown of TRIM31 reduced the proliferation of GC tumor cells and negatively regulated the cell cycle displayed by the decrease of CylclinD1 and CDK2. The decrease of mitochondrial membrane potential was indicated the early stage of cell apoptosis. This tendency was found in TRIM31-knockdown GC cells by using the detection of JC-1. Moreover, the elevated level of ROS was found in TRIM31-knockdown GC cells, suggested oxidative stress may be occurring with the decreasing expression level of TRIM31.

It was found that the sensitivity of cancer therapy was influenced by the expression level of TRIM31. Upregulated TRIM31 suppressed the sensitivity of daunorubicin and promoted the proliferation of AML cell lines by regulating the Wnt/β-catenin pathway [52], and the knockdown of TRIM31 caused DNA damage and radiosensitized colorectal cancer cell lines [51]. In our study, the IC50 of cisplatin and docetaxel were higher in TRIM31-high group, indicated high expression of TRIM31 may contributed to the resistance of cisplatin and docetaxel in GC tumor cells. While the outcomes were opposite in 5-Fu and paclitaxel.

However, limitations were still existed in our study. Firstly, TRIM31 IHC score was submitted to the multivariate Cox regression and a negative result was obtained. This may due to the small samples and a short follow-up visit. Second, specific signaling pathway and molecular mechanism of TRIM31 in cell cycle and stress need to be explored. Moreover, animal experiments were needed to investigate the dynamic role of TRIM31 during the different process of GC tumorigenesis and development in vivo.

In conclusion, our study investigated the whole functional and expression profile and a risk score system based on the TRIM family in GC. Particularly, TRIM31, a member of TRIM family with the predictive value on survival outcome, was chosen for further analysis. The close connection of TRIM31 and tumorigenesis was displayed through the role of TRIM31 in cell cycle and oxidative stress. Further investigation is required to conduct comprehensive pathway analyses in order to elucidate the intricate molecular interaction networks linked to TRIM31.

## Methods and materials

### Data download and processing

RNA-seq transcriptome profiling data and clinicopathological information of GC come from TCGA-STAD (The Cancer Genome Atlas database) and GSE84437 (Gene Expression Omnibus database). The RNA-seq in Fragments Per Kilobase per Million (FPKM) was transformed into Transcripts Per Million (TPM) format and ensemble id was transformed into gene symbol for further analysis. 75 TRIM family members were screened out from GENECARDS database. RNA-Seq profiling data from TCGA-STAD and GSE84437 were merged for calibration and batch correction. The common TRIM family members (TRIMs) were intersected.

### Expression, survival analyses, and gene alteration of TRIMs

The median expression level of TRIMs was calculated for univariate Cox regression analysis between the high and low expression groups. The prognostic network diagram was drawn according to the results of univariate Cox regression analysis. HR > 1 was defined as risk factors, and HR < 1 was favorable factors.

Differential expression analysis of TRIMs was performed using “limma” package in TCGA TPM data. The threshold for defining differential expression is set as |log_2_ (Fold change) |>0.5 and p-adjust value < 0.05. TRIMs of which was expressed differentially which were defined as differentially expressed members (DEMs). The median expression level of DEMs was calculated for K-M survival analysis between the high and low expression groups. Survival curves were drawn for DEMs with p value < 0.05 in K-M survival analysis.

Simple nucleotide variation (SNV) data was downloaded from TCGA database for gene alteration frequency of TRIMs using R “maftools” package. The gene-level copy number data of TCGA-STAD was downloaded from the XENA database for analyzing the copy number alteration frequency.

### Construction of transcription factor (TF)-gene-miRNA and protein-to-protein (PPI) network on TRIMs

The PPI network was based on the construction of the interaction relationship of proteins coded by TRIMs using STRING database with medium confidence (0.400). Cytoscape software (v3.8.2) was used for visualization ranked by the “Degree”. The ENCODE database was chosen for the construction of TF-gene interaction network, with the peak intensity signal < 500 and the predicted regulatory potential score < 1. Additionally, the gene-miRNA interaction network was validated from the miRTarBase database.

### Consensus clustering analysis and risk score system of TRIMs

Consensus clustering analysis was performed based on the expression of TRIMs using R “ConsensusClusterPlus” package. Expression data of TRIMs were obtained from the merged-matrix from TCGA-STAD and GSE84437. The K value with the best stability and clustering effect was selected as the grouping criterion. The Kaplan-Meier (K-M) analysis, single-sample gene set enrichment analysis (ssGSEA) and GSVA analysis were performed between different TRIM clusters. Different expression genes (DEGs) between different TRIM clusters were screened out with the threshold of |Log2 [Fold Change (FC)]| >1 and p value < 0.05. Gene Ontology (GO) and Kyoto Encyclopedia of Genes and Genomes (KEGG) were performed among DEGs.

In order to further investigate the characteristics of TRIMs and to accurately predict prognosis in GC, we aimed to established the risk score system. First, univariate Cox regression analysis on DEGs was performed. Subsequently, DEGs that exhibited significant differences in prognosis were selected for the second consensus clustering analysis to obtained the gene clusters. The K-M analysis were performed between different gene clusters. STAD patients obtained from the merged-matrix were randomly divided in to a train set and a test set. The least absolute shrinkage and selection operator (LASSO) regression was used to perform the variable selection and regularization and the multivariate Cox regression analysis was used for model construction. The risk score was calculated by the following formula: Risk Score = ∑(normalized gene expression * Cox coefficients). Clinicopathological variates and the risk score were included into Cox regression analysis and variates with the p value < 0.05 were used to construct the nomogram. The receiver operating characteristic (ROC) curve and calibration analysis were used to evaluated the predicted ability and accuracy of the model.

### Deep research of TRIM31 with function and prognostic significance

Signaling pathway and cell function enrichment analysis were performed for better understand of the potential effect of TRIM31 on STAD based on the co-expressed genes of TRIM31 and DEGs between TRIM31-high and TRIM31-low groups. Furthermore, the expression level of TRIM31 was regarded as a variate for multivariate Cox regression analysis.

Immune infiltration analysis.

The CIBERSORT analysis was used to explore the correlation between the model constituent genes and immune cells. The indicator of the tumor immune microenvironment (TME), including the stromal score, immune score, and tumor purity were generated with the ESTIMATE algorithm.

### Microsatellite instability and drug sensitive analysis

Microsatellite instability (MSI) status (including microsatellite instability stable (MSS), microsatellite instability -low (MSI-L), and microsatellite instability – high (MSI-H)) of STAD samples were obtained from the TCGA and were performed the related analysis with the risk score. The response to chemotherapy was predicted for each sample based on the Genomics of Drug Sensitivity in Cancer (GDSC) database. The prediction process was implemented by the ‘pRRophetic’ package, in which the half-maximal inhibitory concentration (IC50) of the samples was estimated by ridge regression, and all parameters were set at default values.

### scRNA-seq transcriptome analysis

scRNA-seq data GSM5101015 of GC was downloaded from GSE167297 series. The expression matrix was converted into “Seurat” object and was filtered to reserve cells with the gene content between 200 and 2500. The “PercentageFeatureSet” function was used to calculate the percentage of mitochondrial genes and visualize gene characteristics and sequencing depth. The data was standardized and the top 200 genes with high variation coefficient were extracted. PCA principal component analysis was performed to reduce the dimension of the data, and PC with p-value < 0.05 was selected for further analysis. T-distributed stochastic neighbor embedding (t-SNE) algorithm was used to unsupervised clustering and visualization of clusters. CellMarker 2.0 database was used for cell subgroups’ annotation with the retrieval of marker genes of different PCs. Cell trajectory analysis and cell trajectory difference were performed using the “monocle” R package. Finally, the cell trajectory analysis and cell trajectory difference analysis were performed using the ‘monocle’ package.

### Cell culture and reagents

GES-1, AGS, MKN7, SGC7901, NCI-N87 were purchased from the Cell Bank of Type Culture Collection of the Chinese Academy of Sciences (Shanghai, China) and were all cultured in RPMI 1640 medium (10% fetal bovine serum (FBS), 1% penicillin/ streptomycin) and stably cultured in an incubator (37℃, 5% carbon dioxide).

The following substances or antibodies were used: 3-(4,5-dimethylthiazolyl-2)-2,5-diphenyltetrazolium bromide (MTT; Aladdin: M158055), Crystal violet (Aladdin: C110703), 2′,7′-dichlorodihydrofluorescein diacetate (H2DCFDA; Sigma-Aldrich: D6883), Mitochondrial Membrane Potential Assay Kit (Elabscience Biotechnology Co.,Ltd: E-CK-A301), BeyoClick ™ EdU-488 Cell Proliferation Detection Kit (Beyotime: C0071S), HRP Goat Anti Rabbit IgG (H + L) (Abclonal: AS014), anti-cyclin D1 (Abclonal: A19038), anti-CDK2 (Abclonal: A0094) and anti-GAPDH (Cell Signaling Technology: #5174).

### Establishment of the stable transfected cell line

We bought lentivirus (vector: GV112) (TRIM31-RNAi121410-1 target sequence: GCTCTCAGGATACGAAGSCAT; TRIM31-RNAi121411-1 target sequence: GCCACAGTTGAACGATCTCAA; TRIM31-RNAi121412-1 target sequence: CGTGAATCCAAGGACCACAAA) from Jikai Gene (Shanghai, China) with the goal to create a stable cell line that can lower TRIM31 expression.

Before infecting with lentivirus, place AGS cells (1.2*105/well) in a 6-well plate and incubate in a culture incubator for 24 h. Calculate the required volume of lentivirus per well cell using a MOI (Multiple Infection Index) of 10 and add lentivirus infection enhancer to the culture medium to increase lentivirus infection effectiveness. After 48 h of infection, cells were digested with trypsin to facilitate cell subculture. Puromycin was added to the cell culture media at a concentration of 1 µg/mL to eliminate any uninfected AGS cells. Puromycin must constantly treat cells for one week before collecting cells to detect the expression level of TRM31 protein within the cells and select the appropriate knockdown.

### Cell viability determination

Inoculate 1.3*104/well AGS cells per well in 24-well plates. Stop the cultivation on 1, 3 and 5 days, respectively. Add 500 µL/well MTT (0.5 mg/mL) and incubate for 3 h at 37 °C in the 24-well plates. Add 500 µL DMSO to each well after removing the culture medium to completely dissolve the formaldehyde. Draw a cell growth curve by measuring the solution’s absorbance at 490 nm with a full-function microplate detector (BioTek, USA).

### EdU (5-ethynyl-2’ -deoxyuridine) assay

Inoculate AGS cells (3.3*104/well) for 24–36 h of steady cultivation in the 24-well plates and incubate the EdU working solution for 3 h at 37 ℃. 4% paraformaldehyde was added and fixed for 15 min before incubating the transparent solution for 15 min. After rinsing with PBS, add the Click reaction solution and incubate in the dark for 30 min before staining with Hoechst 33,342 for 15 min. Using an inverted fluorescent microscope (Nikon, Japan) for imaging and photography.

### Migration and colony formation assay

Inoculate AGS cells (3.3*104/well) into 24-well plate and incubate them stably in an incubator. When the cell convergence hits 90%, use the gun head to make a wound on the cell’s surface. PBS was used to clean the cells in each well and to remove any suspended cells. After that, cultivate the cells for 48 h in fresh culture media to examine cell migration.

Inoculate AGS cells (1000/well) into 6-well plate and incubate them for 12 days in a stable incubator. Change the culture media every 3 days. Fix overnight with 4% paraformaldehyde. In each well, incubate 1mL 0.1% crystal violet solution for 2 h before rinsing and drying. Photograph cell clones for examination. Dissolve cell colonies with 30% glacial acetic acid and measure the absorbance of the solution on a full-function microplate detector (BioTek, USA) and perform analysis.

### ROS assay and measurement of mitochondrial membrane potential

AGS cells (3.3*104/well) were seeded in a 24-well plate for stable cultivation for 36 h. We prepared 10 µmol/L H2DCFDA using RPMI 1640 culture medium without FBS. Subsequently, the culture medium was removed and freshly prepared DCFH-DA was added to a 24-well plate and incubated with the cells for 30 min. Fluorescence inverted microscope captured green fluorescence intensity.

JC-1 is often used to detect changes in mitochondrial membrane potential within cells. JC-1 emits strong red fluorescence in normal mitochondria, while green fluorescence is enhanced in damaged mitochondria. JC-1 dye working solution was added to the cell culture plate and then rinsed with buffer. Subsequently, the green fluorescence and red fluorescence intensities were measured using a fluorescence inverted microscope.

### Western blot

Cells should be lysed using Western/IP lysis buffer (Beyotime: P0013) at 4 °C for 60 min. Centrifuge afterwards for 25 min at 12,000 rpm and 4 ° C. Gather the supernatant, measure the protein content, and then boil the sample at 100 °C for 10 min to denaturize the protein. Proteins are separated using the Omni Easy TM Gel before being transferred to polyvinylidene difuoride (PVDF) membranes. The PVDF membrane was first incubated for 2 h at room temperature with a sealing solution, then overnight at 4 °C with anti-TRIM31, anti-CDK2, anti-cyclinD1, and anti-GAPDH, and finally for 2 h at room temperature with HRP Goat Anti Rabbit IgG (H + L). Protein expression is discovered using an Electrochemical Luminescence (ECL) detection kit.

### Tissue microarray chips and immunohistochemistry (IHC) staining

Tissue microarray chips consisted 97 STAD patients’ samples and 83 paracancerous stomach tissue samples were purchased from Shanghai Qutdo Biotech Company (Shanghai, China). Tissue microarray chips was incubated with monoclonal rabbit anti-human TRIM31 (dilution 1:1500, Proteintech, Wuhan, China) after formalin fixation. IHC score = staining intensity score × percentage of positive staining. K-M analysis was performed between TRIM31-high and TRIM31-low groups. Age, sex, tumor size, pathological grade, T stage, N stage, M stage and TRIM31 expression were included in the univariate and multivariate Cox regression analysis. The study protocol was approved by the Shanghai Qutdo Biotech Company Ethics Committee (NO. YB M-05-02).

### Electronic supplementary material

Below is the link to the electronic supplementary material.

Supplementary Material 1

Supplementary Material 2

Supplementary Material 3

Supplementary Material 4

Supplementary Material 5

Supplementary Material 6

Supplementary Material 7

Supplementary Material 8

Supplementary Material 9

## Data Availability

No datasets were generated or analysed during the current study.
