# Supplementary material for: An investigation of the molecular characterization of the tripartite motif (TRIM) family and primary validation of TRIM31 in gastric cancer
Source: Hum Genomics. 2024 Jul 9;18:77. doi: 10.1186/s40246-024-00631-7 (PMC11232234; doi:10.1186/s40246-024-00631-7)
Supplement: Supplementary file 1 — Supplementary Material 1 [file 40246_2024_631_MOESM1_ESM.docx]

**Supplemental figure legends**

**Supplemental figure 1 and 2.** K-M analysis of 44 DEMs that were significantly correlated with the OS. ns, p≥0.05; *p< 0.05; **p<0.01; ***p<0.001.

**Supplemental figure 3.** The delta area, consistent cumulative distribution function (CDF) plot and consensus matrix legend showed the best choice of the K value in the first (A) and the second (B) consensus clustering analysis, respectively. (C) CFMs differentially expressed in gene clusters A and B. (D) The LASSO regression analysis to filtrate hub genes among DEGs. The ROC curve with AUC of the predicted model including risk score in train set (E) and test set (F) at 1, 3, and 5-year. ns, p≥0.05; *p< 0.05; **p<0.01; ***p<0.001.

**Supplemental figure 4.** The risk score, survival time and expression level of hub genes in high-risk and low-risk groups in train cohort (A) and test cohort (B). K-M analysis between high-risk and low-risk groups in train cohort (C) and test cohort (D). (E) CFMs differentially expressed in high-risk and low-risk groups. (F)Scatterplot of immune cells with significant statistical difference with risk scores. ns, p≥0.05; *p< 0.05; **p<0.01; ***p<0.001.

**Supplemental figure 5.** (A) The distribution of clinical characteristics of the samples in the TRIM31-high and TRIM31-low expression groups was analyzed by chi-square test. (B)The network of TF-TRIM31 and miRNA-TRIM31. (C)The PPI network of co-expressed genes of TRIM31. (D) GO and KEGG analyses on co-expressed genes of TRIM31. (E) GSEA analysis on DEGs between TRIM31-high and TRIM31-low groups. (F) Scatterplot of immune cells correlating with TRIM31 obtained by Spearman correlation analysis. (G) There was a significant difference in stromal score between the TRIM31-high and TRIM31-low expression groups. ns, p≥0.05; *p< 0.05; **p<0.01; ***p<0.001.
