# Supplementary material for: An investigation of the molecular characterization of the tripartite motif (TRIM) family and primary validation of TRIM31 in gastric cancer
Source: Hum Genomics. 2024 Jul 9;18:77. doi: 10.1186/s40246-024-00631-7 (PMC11232234; doi:10.1186/s40246-024-00631-7)
Supplement: Supplementary file 2 — Supplementary Material 2 [file 40246_2024_631_MOESM2_ESM.docx]

**Supplementary table 1. Clinical characteristics of STAD samples in tissue microarray chips.**

| **Characteristics** | **Univariate analysis** | |  | **Multivariate analysis** | |
| --- | --- | --- | --- | --- | --- |
|  | Hazard ratio (95% CI) | P value |  | Hazard ratio (95% CI) | P value |
| **IHC score of TRIM31** |  |  |  |  |  |
| low | Reference |  |  | Reference |  |
| high | 1.642 (1.003 - 2.689) | **0.049** |  | 0.947 (0.555 - 1.614) | 0.840 |
| **Sex** |  |  |  |  |  |
| Male | Reference |  |  |  |  |
| Female | 1.070 (0.617 - 1.857) | 0.809 |  |  |  |
| **Age** | 1.030 (1.004 - 1.057) | **0.022** |  | 1.023 (0.996 - 1.051) | 0.102 |
| **T stage** |  |  |  |  |  |
| T1 | Reference |  |  | Reference |  |
| T2 | 1.441 (0.373 - 5.574) | 0.596 |  | 1.242 (0.318 - 4.843) | 0.755 |
| T3 | 3.515 (1.076 - 11.486) | **0.037** |  | 2.371 (0.702 - 8.003) | 0.164 |
| T4 | 9.291 (2.754 - 31.351) | **< 0.001** |  | 5.546 (1.537 - 20.009) | **0.009** |
| **N stage** |  |  |  |  |  |
| N0 | Reference |  |  | Reference |  |
| N1 | 0.680 (0.266 - 1.737) | 0.420 |  | 0.834 (0.323 - 2.152) | 0.708 |
| N2 | 1.547 (0.788 - 3.040) | 0.205 |  | 1.361 (0.674 - 2.749) | 0.390 |
| N3 | 2.793 (1.492 - 5.228) | **0.001** |  | 2.097 (1.061 - 4.145) | **0.033** |
| **Pathological grade** |  |  |  |  |  |
| Well differentiated | Reference |  |  |  |  |
| Moderately differentiated | 1.468 (0.440 - 4.894) | 0.532 |  |  |  |
| Poorly differentiated | 2.237 (0.691 - 7.247) | 0.179 |  |  |  |
| **Helicobacter pylori** |  |  |  |  |  |
| positive | Reference |  |  |  |  |
| negative | 0.989 (0.571 - 1.714) | 0.970 |  |  |  |
| unknown | 1.088 (0.583 - 2.028) | 0.791 |  |  |  |
