# Supplementary figures and images for: An investigation of the molecular characterization of the tripartite motif (TRIM) family and primary validation of TRIM31 in gastric cancer
Source: Hum Genomics. 2024 Jul 9;18:77. doi: 10.1186/s40246-024-00631-7 (PMC11232234; doi:10.1186/s40246-024-00631-7)

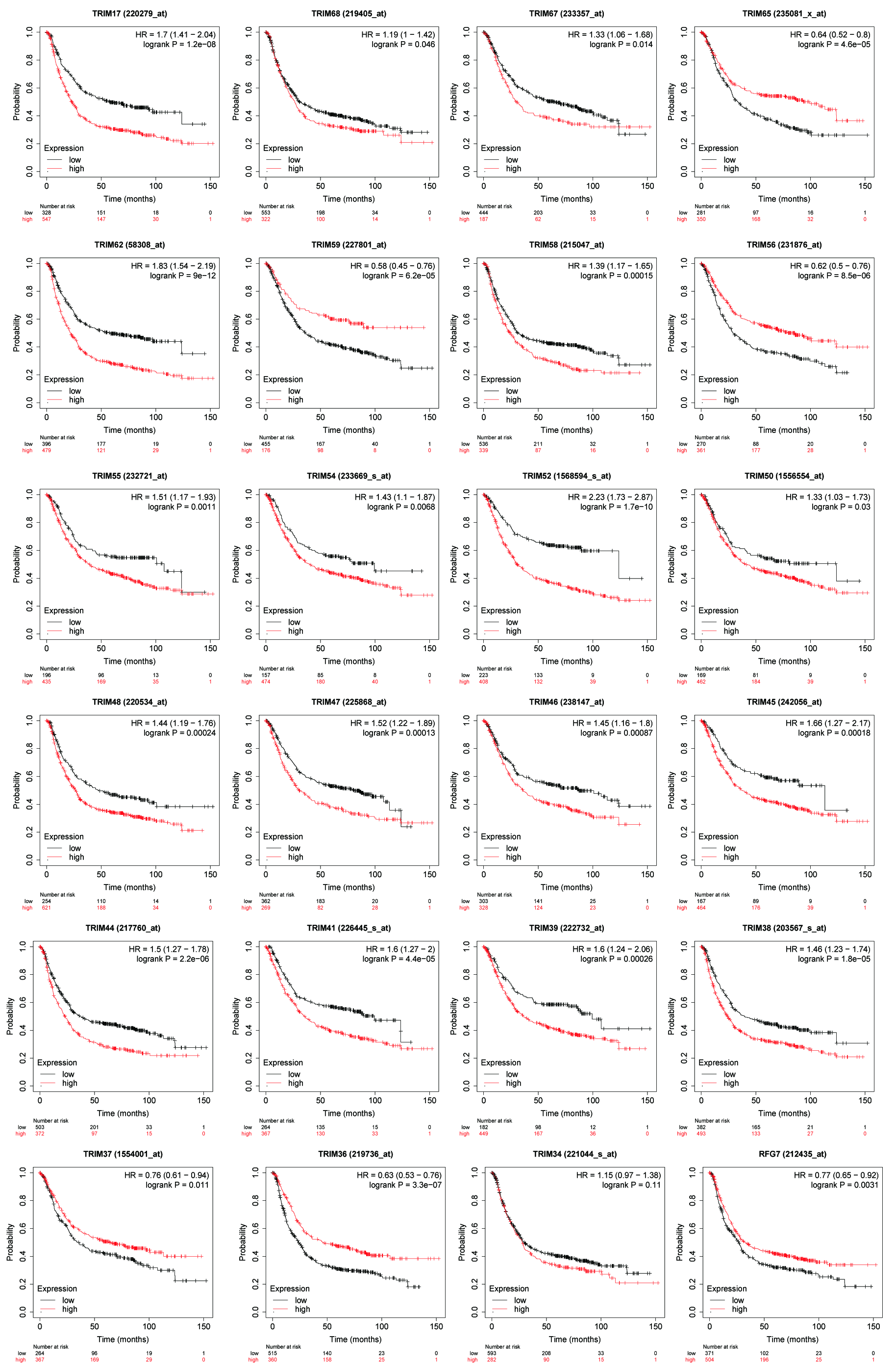

Supplement: Supplementary file 3 — Supplementary Material 3 [file 40246_2024_631_MOESM3_ESM.tif]

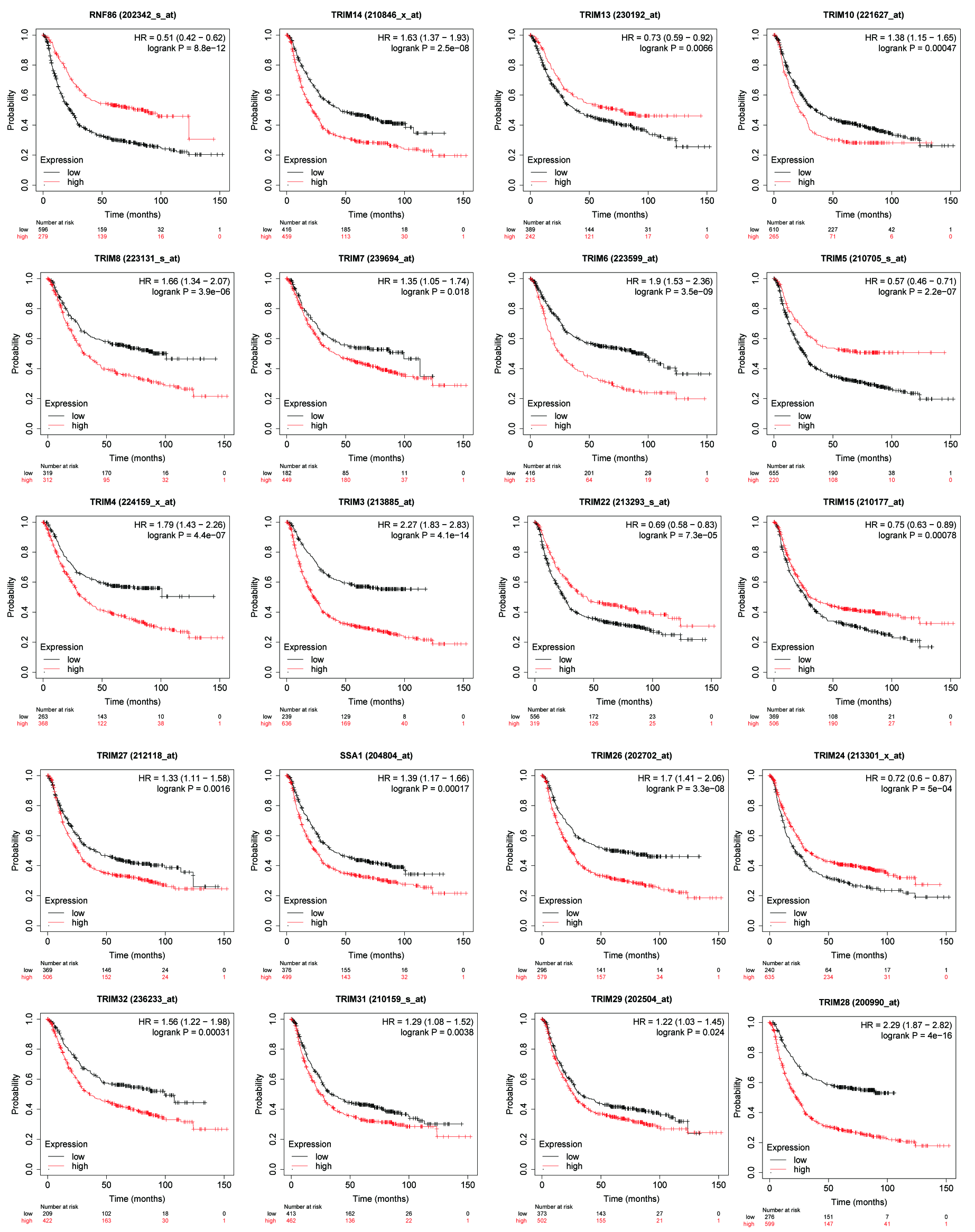

Supplement: Supplementary file 4 — Supplementary Material 4 [file 40246_2024_631_MOESM4_ESM.tif]

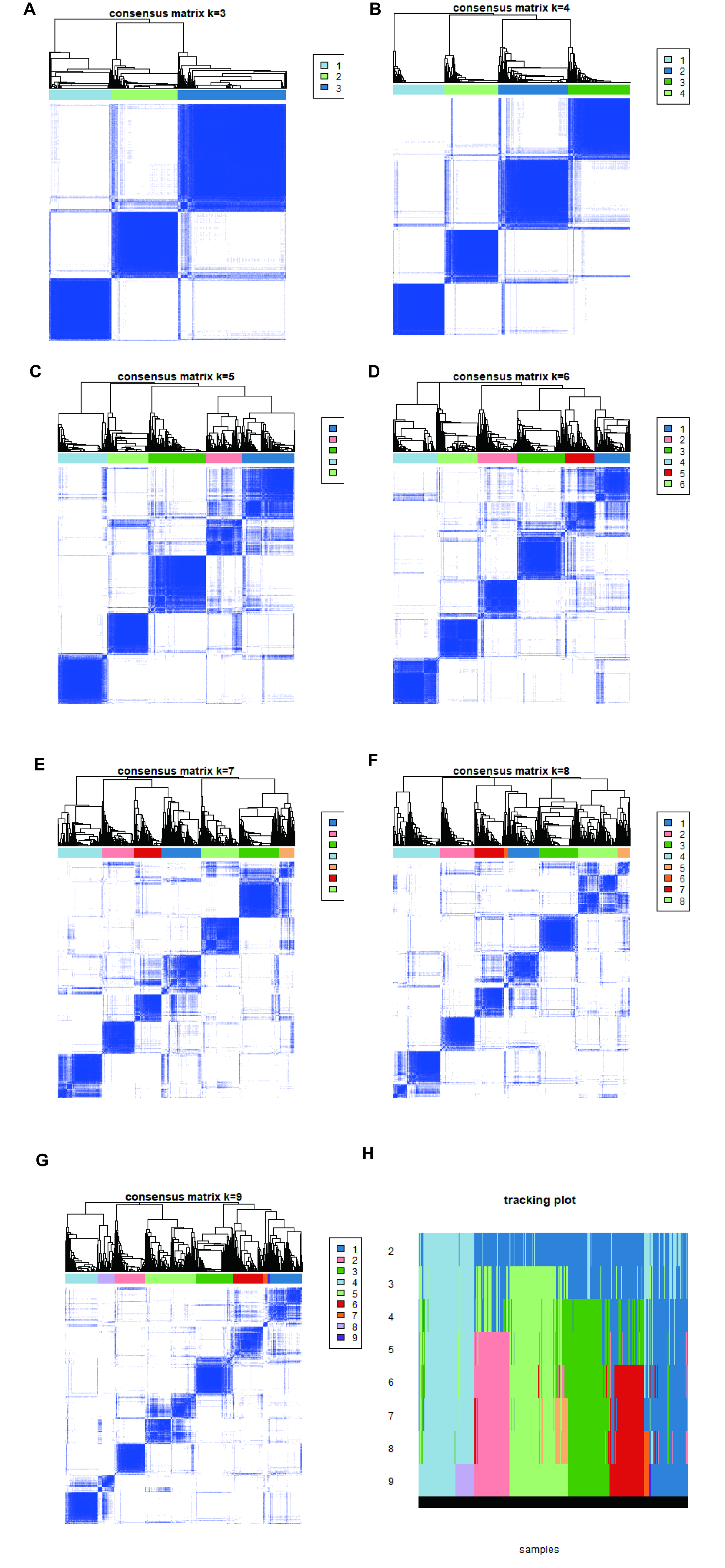

Supplement: Supplementary file 5 — Supplementary Material 5 [file 40246_2024_631_MOESM5_ESM.tif]

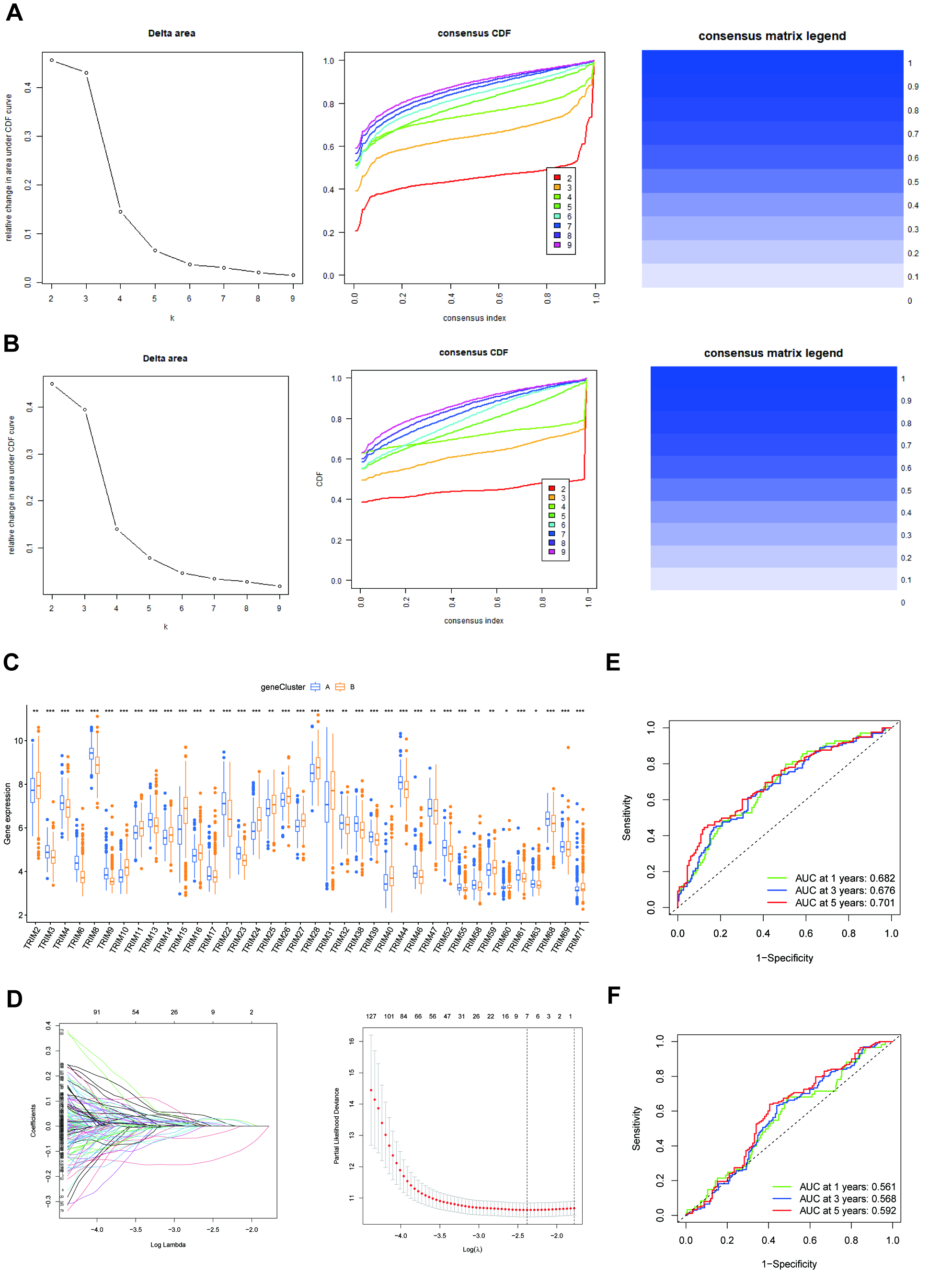

Supplement: Supplementary file 6 — Supplementary Material 6 [file 40246_2024_631_MOESM6_ESM.tif]

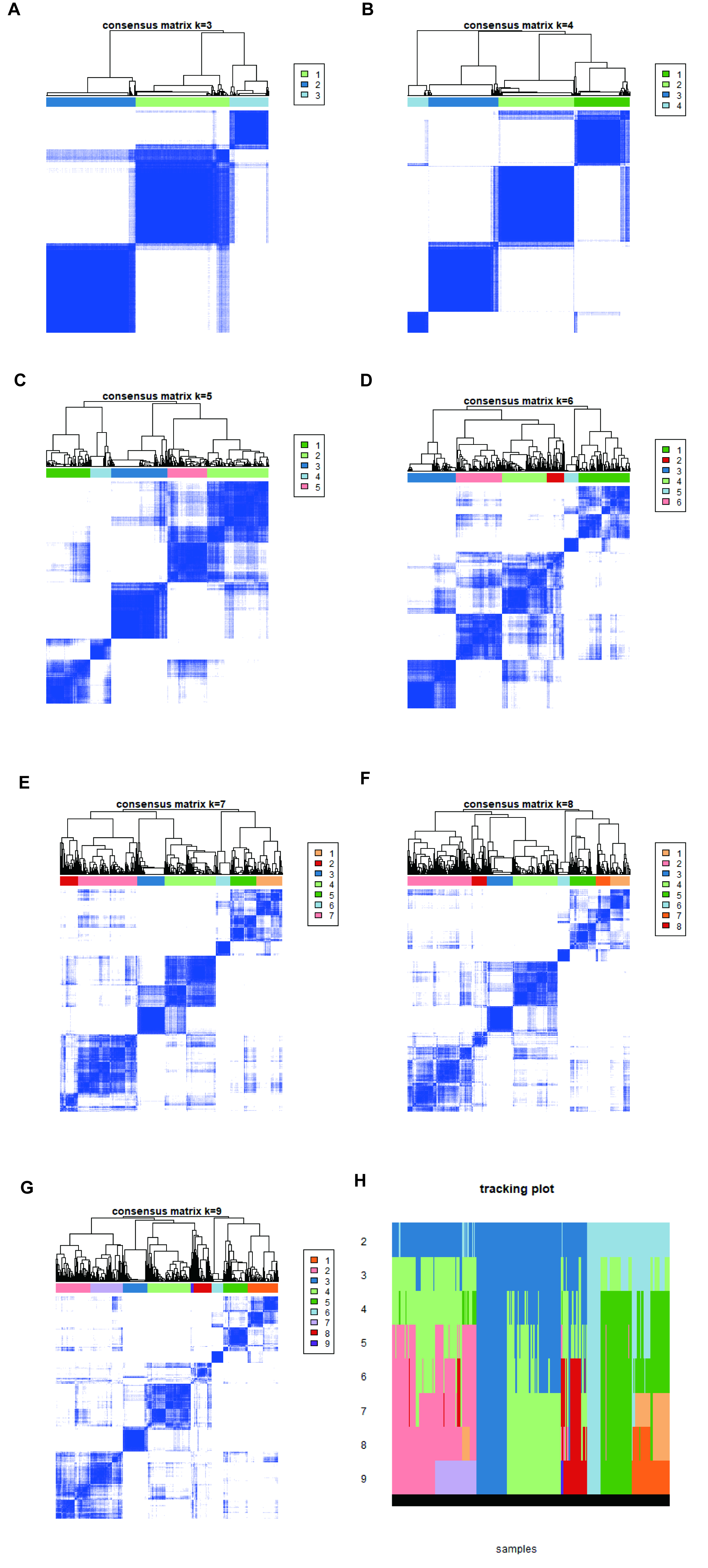

Supplement: Supplementary file 7 — Supplementary Material 7 [file 40246_2024_631_MOESM7_ESM.tif]

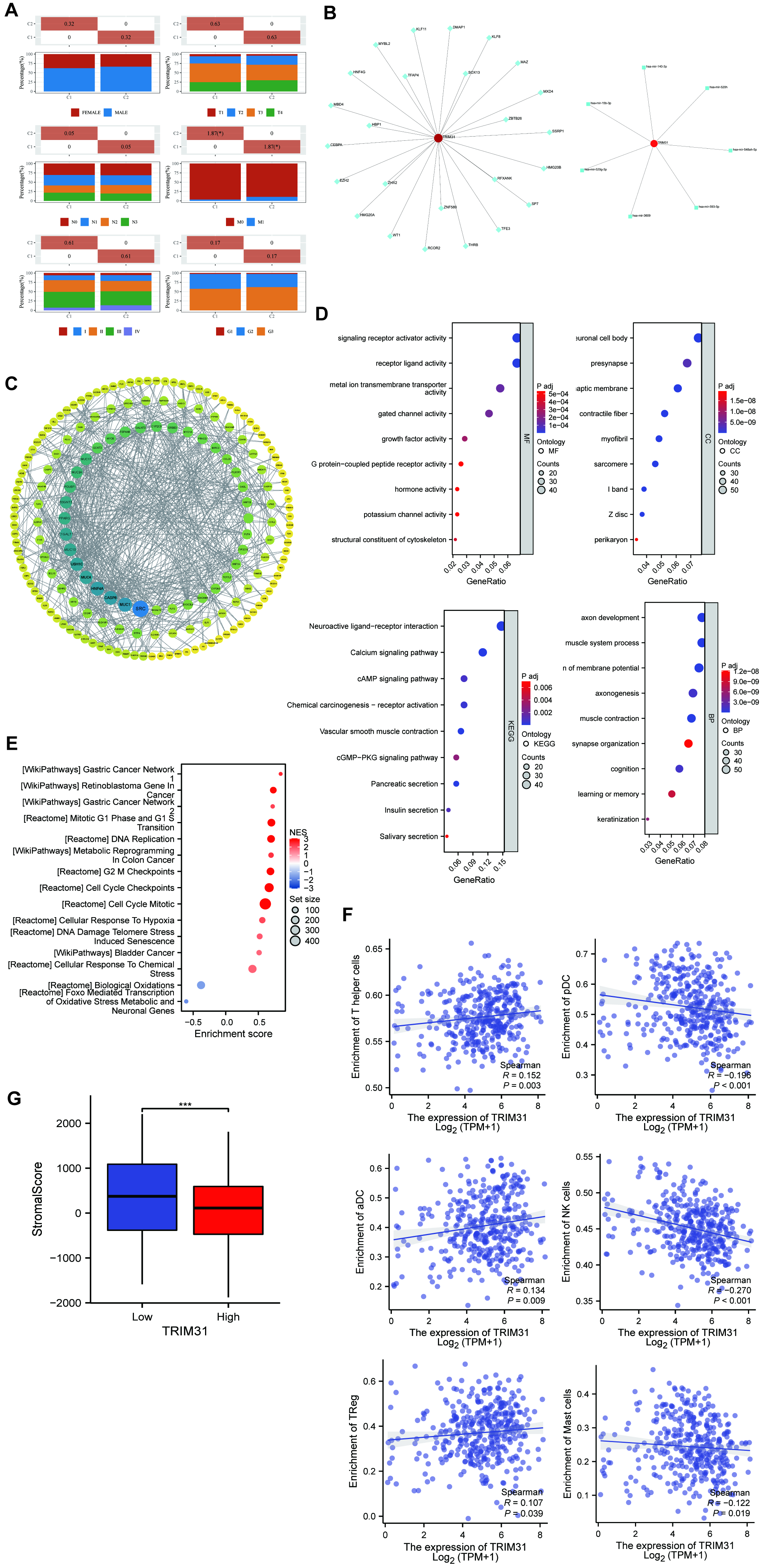

Supplement: Supplementary file 8 — Supplementary Material 8 [file 40246_2024_631_MOESM8_ESM.tif]

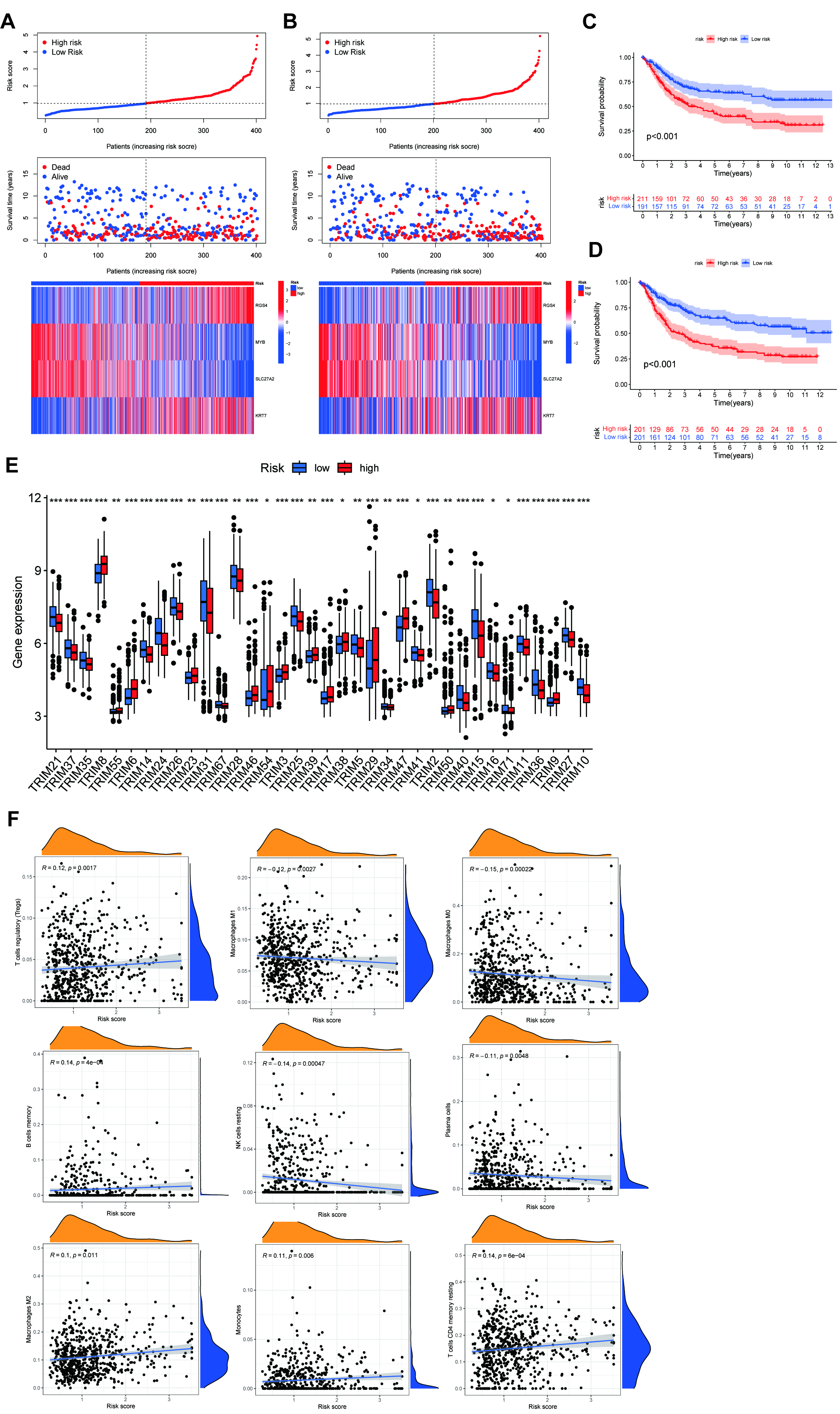

Supplement: Supplementary file 9 — Supplementary Material 9 [file 40246_2024_631_MOESM9_ESM.tif]
